# Application of Coagulation–Membrane Rotation to Improve Ultrafiltration Performance in Drinking Water Treatment

**DOI:** 10.3390/membranes11080643

**Published:** 2021-08-21

**Authors:** Hongjian Yu, Weipeng Huang, Huachen Liu, Tian Li, Nianping Chi, Huaqiang Chu, Bingzhi Dong

**Affiliations:** 1 College of Environmental Science and Engineering, Tongji University, Shanghai 200092, China; 1853855@tongji.edu.cn (H.Y.); oeleo@tongji.edu.cn (W.H.); liuhuachen00@163.com (H.L.); chuhuaqiang@tongji.edu.cn (H.C.); dbz77@tongji.edu.cn (B.D.); 2 Key Laboratory of Yangtze River Water Environment, Ministry of Education, Tongji University, Shanghai 200092, China; 3 State Key Laboratory of Pollution Control and Resource Reuse, Tongji University, Shanghai 200092, China; 4 School of Municipal and Geomatics Engineering, Hunan City University, Yiyang 413000, China; chinianping@163.com

**Keywords:** membrane rotation, coagulation, UF, dissolved organic matter, membrane fouling control, hydrodynamic shear stress

## Abstract

The combination of conventional and advanced water treatment is now widely used in drinking water treatment. However, membrane fouling is still the main obstacle to extend its application. In this study, the impact of the combination of coagulation and ultrafiltration (UF) membrane rotation on both fouling control and organic removal of macro (sodium alginate, SA) and micro organic matters (tannic acid, TA) was studied comprehensively to evaluate its applicability in drinking water treatment. The results indicated that membrane rotation could generate shear stress and vortex, thus effectively reducing membrane fouling of both SA and TA solutions, especially for macro SA organics. With additional coagulation, the membrane fouling could be further reduced through the aggregation of mediate and macro organic substances into flocs and elimination by membrane retention. For example, with the membrane rotation speed of 60 r/min, the permeate flux increased by 90% and the organic removal by 35% in SA solution, with 40 mg/L coagulant dosage, with an additional 70% increase of flux and 5% increment of organic removal to 80% obtained. However, too much shear stress could intensify the potential of fiber breakage at the potting, destroying the flocs and resulting in the reduction of permeate flux and deterioration of effluent quality. Finally, the combination of coagulation and membrane rotation would lead to the shaking of the cake layer, which is beneficial for fouling mitigation and prolongation of membrane filtration lifetime. This study provides useful information on applying the combined process of conventional coagulation and the hydrodynamic shear force for drinking water treatment, which can be further explored in the future.

## 1. Introduction

Ultrafiltration (UF) membranes are widely used in drinking water treatment [1,2,3] due to the advantages of easy operation and superior effluent quality with moderate energy consumption [4,5]. However, membrane fouling is still the main obstacle of UF to constrain its development. In drinking water treatment systems, the dissolved organic matter (DOM) are key membrane fouling contributors [6,7,8]. DOM can block the membrane pores or form a gel layer on the membrane surface to limit the filtration pathway or increase the filtration resistance, resulting in a reduced permeability that requires frequent cleaning or even membrane replacement [9,10,11].

To reduce membrane fouling, many kinds of methods have been tried, among them, vibrating membranes are considered as one of the most useful technologies and has attracted great concern recently [12,13]. During vibration, a relative movement between the membrane and the neighboring fluid is generated; thus shear stress could be produced [14,15,16], then the foulants can be destroyed and swept away from the membrane surface [15,17]. The membrane vibration could be either longitudinal or transverse, in the former with the membrane parallel to the vibration direction, while in the latter with the membrane perpendicular to the vibration direction. The vibration can be implemented through reciprocating or rotating the membranes. Both operations have been applied in drinking water [18,19] and wastewater treatment [20,21,22], and significant membrane fouling was reduced. Li et al. studied the reciprocating movement of hollow fiber membranes with both longitudinal and transverse motions in yeast and Bentonite suspensions, and they found that the transverse vibration of hollow fiber membranes could be more effective in Bentonite suspensions than in yeast suspensions for membrane fouling reduction, not only due to the high shear stress generation but also the secondary flows production [23]. Kim et al. investigated the membrane rotation for anaerobic wastewater treatment, and they found that with membrane rotation, the shear force and the mass transfer were enhanced, thus, the membrane fouling could be greatly reduced [24]. As compared with the membrane reciprocation, the membrane rotation can be more effective since sinusoidal shear stress could be generated by the reciprocating membrane while constant shear force by the rotating membranes. Moreover, vortex could be also generated in a membrane rotation reactor when the fluid was mobilized as the membrane module in a circular motion continuously.

In the hydrodynamic system, the particle size of the influent has a great impact on the shear stress and the associated membrane performance. It is reported that large particles could produce high shear force in a hydrodynamic system [25,26] and can be easily prevented by the membranes [27,28]. However, in the low-pressure membrane filtration system, the membrane performance was determined by integrated conditions, including the feed characteristics, the membrane properties, and the hydrodynamic conditions [29]. Generally, the colloids with larger molecular weight and lower viscosity can induce less membrane fouling in a stronger fluid motion system, while the organic substances with lower molecular weight and higher viscosity in a stable environment may stick on the membrane surface or block the membrane pores, resulting in a severer membrane fouling and a lower permeability [30,31,32]. For example, Gao et al. found that the hydrophilic macro-molecules could form a compact, thin foulant layer with high roughness on the hydrophilic UF membrane and hydrophilic micro-molecule substances could create a compact thin foulant layer with low roughness on the UF membrane [9]. Li et al. separated the yeast suspensions to the extracellular polymeric substance (EPS) and washed yeast, and they observed that the EPS could induce a much higher filtration resistance even with a much lower concentration due to its higher viscosity and lower particle size [23]. In comparison, Fallahianbijan et al. found that the membrane pore structure was key to membrane filtration performance [33].

Besides membrane vibration, coagulation is widely used in drinking water treatment to reduce UF membrane fouling and enhance organic removal [18,34]. In the coagulation process, coagulants, such as Al (III) and Fe (III) compounds, were added to the influent. The colloids, usually with a negative charge, can be combined with the positive trivalent ions to form flocs, which can reduce its stability and settle down in the subsequent sedimentation process. With the reduction of the colloid in the coagulation process, the influent DOM concentration for the followed UF thus can be much lowered [34]. In the coagulation process, the dosage amount is a very important parameter since insufficient coagulant dosage cannot aggregate the DOM to form flocs, while too many coagulants could make the colloid stable again and cannot induce effective precipitation resulting in a reduced organic removal. The flocs sizes are key to coagulation. Generally, large flocs had an efficient precipitation ability, while small flocs were difficult to settle down. In addition to the coagulant dosage amount, the hydrodynamic condition is also an important issue for effective coagulation [34,35]. The flocs were easy to precipitate in a relatively stable environment, while they may be broken down in an environment with strong fluid motion since flocs are relatively loose [34].

The combination of membrane vibration and coagulation thus can be a potentially useful technique for UF pretreatment. The coagulation could aggregate the DOM to form large particle flocs while the membrane vibration could produce a high shear force to eliminate the particle flocs [34,35]. However, the flocs may be destroyed when too much shear stress was applied, resulting in the ineffectiveness of the membrane fouling control and the deterioration of effluent quality. However, such studies of membrane filtration and its associated organic removal have not been evaluated in a rotating membrane system as far as we are aware.

The objective of this study is to operate a membrane rotation system coupled with coagulation for DOM removal and membrane fouling control. Two influent organic substances with typical macro (i.e., sodium alginate, SA) and micro molecules (i.e., tannic acid, TA) were adopted. The flux performance and the organics removal ratios with different rotating speeds and coagulant dosages were examined comprehensively. Moreover, the floc size and the membrane morphology during the coagulation–membrane rotation process were also investigated such that the combined effect intensifying the membrane performance could be further explored. Before detailed results were reported, the experimental procedures were presented first in the following.

## 2. Materials and Methods

### 2.1. Experimental Setup and Operation Procedures

**Figure 1:**
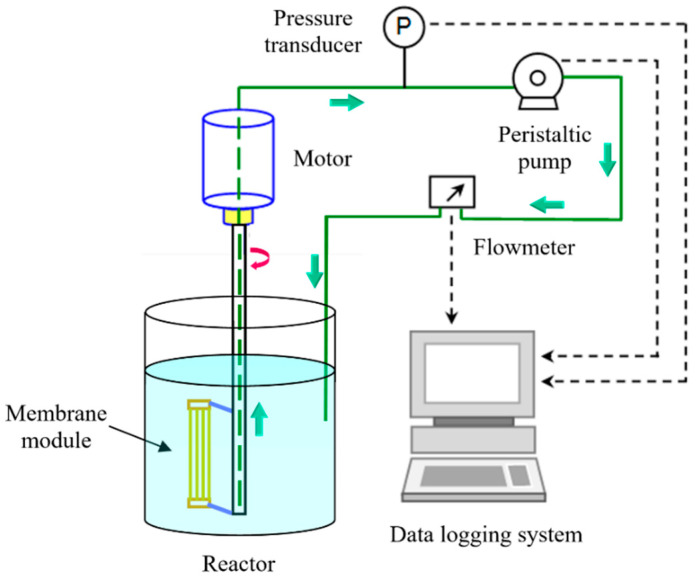
Schematic diagram of the experimental setup.

A schematic diagram of the membrane rotation setup is shown in Figure 1. The setup included the rotation system and the permeate measurement equipment. The test reactor was made of Perspex with a size of 450 mm (Φ) × 300 mm (H). The membrane module was driven by a motor with adjustable rotation speeds. The center of the membrane module was 70 mm away from the axis of the membrane module. The permeate was driven by a peristaltic pump connecting to the data logging system in order to obtain a constant pressure operation mode, and it was sent back to the reactor to maintain a constant volume. The experiment was operated with constant trans-membrane pressure (TMP) of 50 ± 1 kPa, while the permeate flowrate was recorded automatedly with a digital flowmeter with a selected time interval.

### 2.2. Hollow Fibre Membrane Module

PVDF hydrophilic UF hollow fibers from the Dow Chemical Co. with inner/outer diameter of 1.0 mm/1.3 mm, molecular weight cut-off (MWCO) of 150,000 Da, and pore size of 0.03 µm were adopted in the experiment. The module consists of 25 fibers with a length of 185 mm with both ends fixed with a water container using Araldite epoxy; then the water container was connected to the motor axis with screws and nuts.

### 2.3. Feed Solutions and Coagulants

**Figure 2:**
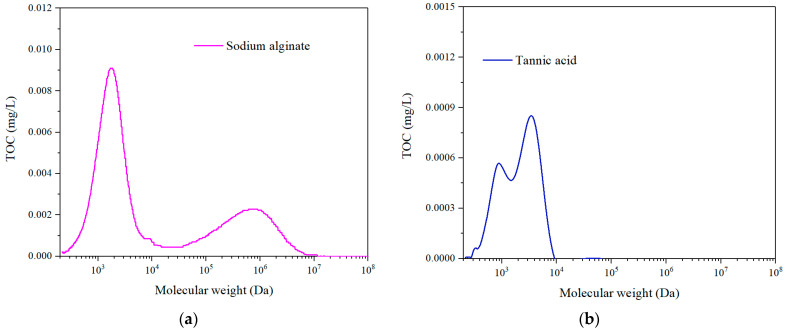
Molecular weight distribution of (**a**) sodium alginate and (**b**) tannic acid.

In this study, two kinds of feed solutions, sodium alginate (SA) and tannic acid (TA) with a concentration of 20 mg/L were mixed with tap water as the feed solutions. They represent the macro and micro organic feeds, respectively, with the molecular weight distribution shown in Figure 2 and product information in Table 1. The coagulant of polyaluminum chloride (PAC) (with detailed product information listed in Table 1) with different concentrations of 20, 30, and 40 mg/L was adopted to examine the coagulation effect on the fouling alleviation of rotating membrane to remove dissolved organics.

### 2.4. Membrane Filtration Analysis

The membrane filtration flux, *J*, can be calculated with Equation (1) as

(1) $$J=\frac{Q}{S}$$

where *Q* is the permeate flowrate recorded from the digital flowmeter, and *S* is the effective membrane surface area. In this study, the specific flux *J*/*J*_0_ was adopted to evaluate the membrane rotation performance, with *J*_0_ the initial membrane filtration flux.

### 2.5. Theory of Membrane Rotation

Earlier Al-Akoum et al. calculated the maximum shear rate, $\gamma_{m}$, of a rotating disk at the periphery given by [36]

(2) $$\gamma_{m}=2^{0.5}d{\left(\pi F\right)}^{0.5}\upsilon^{-0.5}$$

where *d* is the membrane displacement at the periphery, *F* the frequency, and *υ* the permeate viscosity. Since in a rotating hollow fiber reactor, the membrane displacement is identical along the fiber length, thus, such an equation can be also adopted in the current membrane rotation system.

### 2.6. Analytical Methods

The dissolved organic carbon (DOC) concentration was measured with a total organic carbon (TOC) analyzer (TOC-VCPH, Shimadzu, Japan). The molecular weight distribution of the influent and the permeate samples were examined with a high-pressure size exclusion chromatography (HPSEC) method with both DOC detector (TOC, Sievers 900 Turbo TOC) and UV detector (UVA, Waters 2489). The particle size distribution of the SA and TA with different dosages of coagulants before and after membrane rotation was measured with a Mastersizer (MS3000). The images of the surface of the membranes were observed using a scanning electron microscope (SEM, Philips XL30, Amsterdam, The Netherlands).

## 3. Results and Discussion

### 3.1. Effect of Membrane Rotation to Improve UF Performance

**Figure 3:**
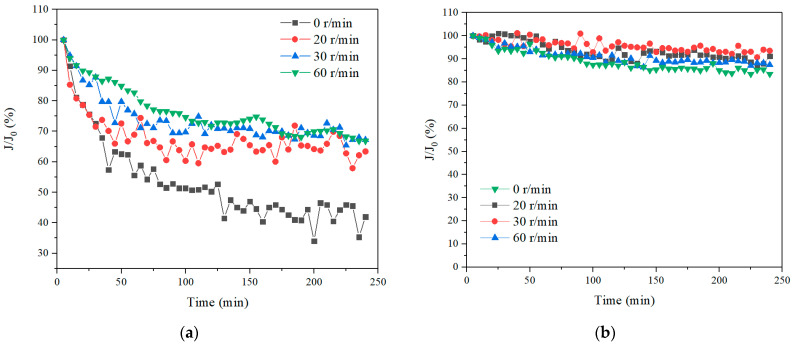
Permeate flux with different rotation speeds, (**a**) SA, and (**b**) TA.

The specific permeate flux performance of the membrane module in both SA and TA solutions was examined first and the results are shown in Figure 3. The flux dropped significantly in SA solution without hollow fiber rotation. With the increase of the membrane rotation speed, the flux improved as well. With 60 r/min rotation, the flux increased by 90% at the end of 4 h filtration. This suggested that the membrane rotation was very effective for flux enhancement in SA solution. In contrast, the flux only decreased slightly for 4 h filtration without membrane rotation in TA solution. While with membrane rotation, the flux increased as well. This indicated that the TA solution induced less membrane flux reduction than the SA solution, and the membrane rotation were effective for the flux improvement for both SA and TA solutions. The phenomenon of better filtration performance of large molecules than small organics was also observed in stationary UF and forward osmosis (FO) operations [7,37].

**Figure 4:**
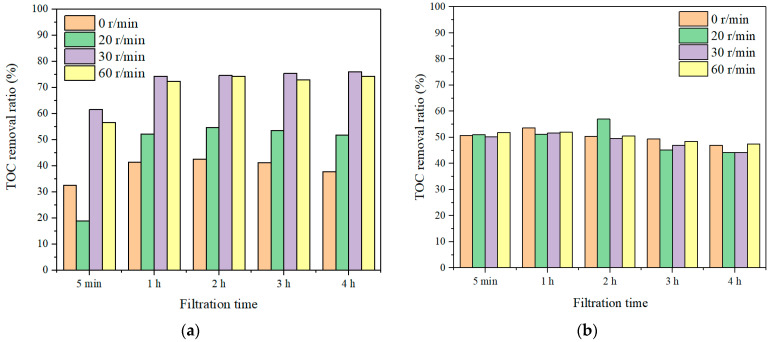
TOC removal ratios with different rotation speeds, (**a**) SA, and (**b**) TA.

The TOC of the permeate with different rotation speeds and different sampling intervals for both SA and TA solutions were measured and the results are presented in Figure 4. In the SA solution, the TOC removal was very low, i.e., ~40% without membrane rotation. With 20 r/min membrane rotation, the TOC removal ratio increased to ~55%. When the membrane rotation speed increased to 30 r/min, the TOC removal ratio increased significantly to ~75%. With the additional increase of the membrane rotation speed to 60 r/min, the TOC removal ratio maintained nearly the same as that with 30 r/min rotation. In contrast, in TA solution, the TOC removal ratios maintained nearly the same, ~50%, no matter how much rotation speeds were applied. This suggested that the membrane rotation was more effective for macro molecule removal, which was probably due to a higher shear force being produced with the macro molecules under rotation, leading to less accumulation of foulants approaching the membranes. Such phenomenon of high removal of large molecules was also observed in UF studies of natural organic matter (NOM) removal [38,39]. In addition, it can be also found that the TOC removal ratio increased in the first hour of filtration, then it maintained constant for the rest 3 h filtration in SA solution, while it kept almost constant (~50%) for all 4 h filtration in TA solution. Such performance was different from dead-end UF filtration, where the foulants could form a cake layer and constrain the pathway of organics resulting in an increased filtration resistance and organics removal [40,41]. This suggested that the rotating membrane could generate the shear stress to prevent membrane fouling and it is also effective for organic removal for long time operations.

**Figure 5:**
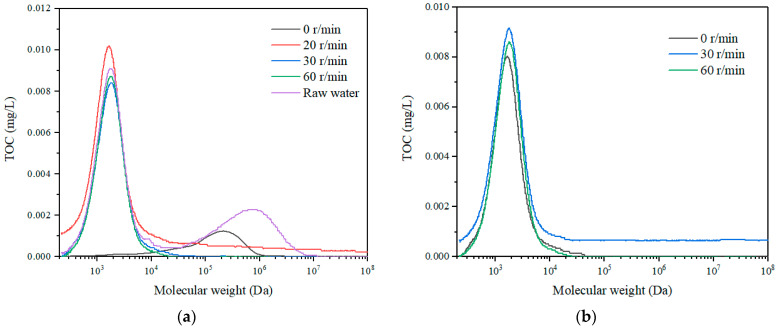
Molecular weight distribution (TOC detection) with different rotation speeds in SA solution, (**a**) 1 h, and (**b**) 4 h.

**Figure 6:**
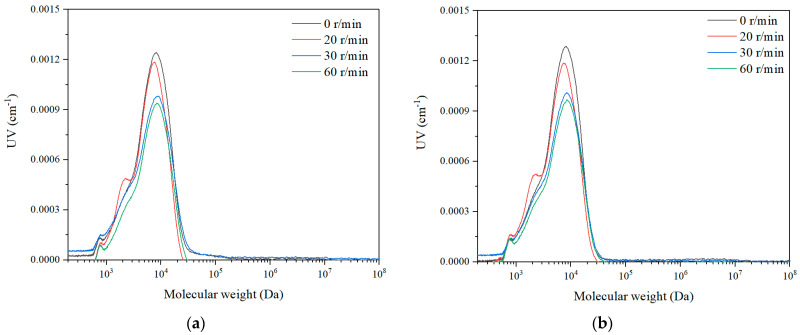
Molecular weight distribution (UV detection) with different rotation speeds in SA solution, (**a**) 1 h, and (**b**) 3 h.

The molecular weight distribution of SA solution with both TOC detection (Figure 5) and UV detection (Figure 6) with different rotation speeds were examined. The results indicated that there were three obvious peaks for the HPSEC curve of SA, i.e., 1000–10,000 Da, 10,000–100,000 Da, and 100,000–1,000,000 Da. Since the hollow fiber membrane has an MWCO of 150,000 Da, it could remove part of the macro molecules with a molecular weight of 100,000–1,000,000 Da due to the sieving effect, while with the additional rotation, the peak intensity of the macro molecules with a molecular weight of 100,000–1,000,000 Da reduced completely. This suggested that the membrane rotation was effective in removing large molecule organics. With the membrane rotation, the peak intensity of the micro molecule organic matter with molecular weight of 1000–10,000 Da only dropped slightly, which indicated that the membrane rotation could eliminate very limited small organic substances with the removal, probably due to adsorption. It can be also observed that with the increase of the rotation speeds, the peak intensities for both macro and micro molecules reduced, which suggested that the membrane rotation could intensify the removal of organics. Moreover, the molecular weight distributions of the organics after membrane rotation for different permeate durations were nearly the same, thus, the membrane rotation could be effective for organics removal for long time operations.

### 3.2. Effect of Coagulation–Membrane Rotation to Improve UF Performance

#### 3.2.1. Effect of Coagulation Dosages on Coagulation–Membrane Rotation Performance

**Figure 7:**
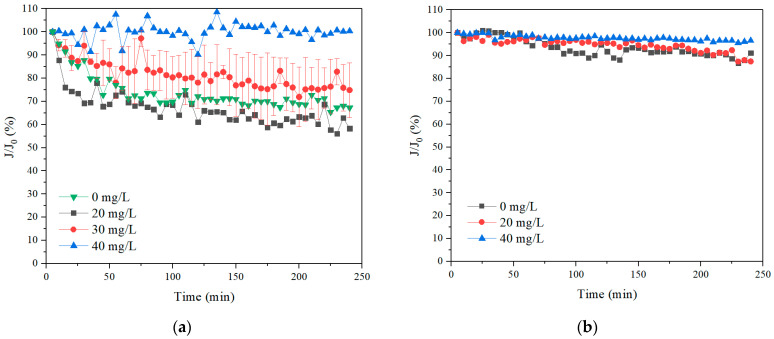
Permeate flux with a rotation speed of 30 r/min and different coagulant dosages, (**a**) SA, and (**b**) TA.

The specific permeate flux performance of the membrane module for different dosages of coagulants at the rotation speed of 30 r/min in both SA and TA solutions were examined and the results are shown in Figure 7. With a coagulant dosage of 20 mg/L, the permeate flux was nearly the same as that without coagulation for both SA and TA solutions. While the permeate flux dropped significantly in the SA solution, and it decreased slightly in TA solution for the 4 h filtration with a low dosage of coagulants, which was probably due to the different molecular weight distributions of SA and TA (in Figure 2). When the coagulant dosage increased to 40 mg/L, the permeate flux rose greatly, and kept almost the same as the initial flux for both SA and TA solutions, that is, almost no fouling. Ma et al. studied the low dosage of aluminum chloride coagulant on the pretreatment of UF by humic acid (HA), bovine serum albumin (BSA), and their mixtures [42]. They found that the membrane filtration performance was greatly affected by the coagulant dosage as well as the pH. At the pH of 7.0 and 8.0, the membrane filtration performance dropped except for the micro organics with high dosages of coagulant, while it also decreased except for all the organics with high dosages of coagulant at the pH of 6.0. They attributed the performance to the critical dosage of coagulant, such that below the critical point, the membrane filtration mode was at the pore blockage resulting in a reduced permeate flux, while beyond the critical dosage, macromolecule cake layer was formed, leading to membrane fouling reduction. Our results suggested that no matter the dosage of coagulant, the Coagulation–Membrane rotation was effective to reduce membrane fouling.

**Figure 8:**
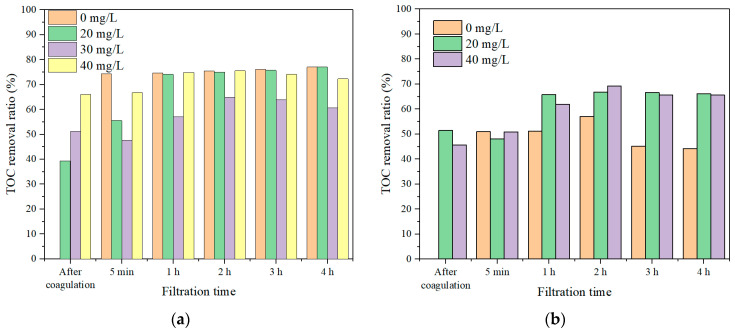
TOC removal ratios with a rotation speed of 30 r/min and different coagulant dosages, (**a**) SA, and (**b**) TA.

The TOC removal ratios with different dosages of coagulants at the rotation speed of 30 r/min for both SA and TA solutions were investigated and the results are shown in Figure 8. The direct coagulation has only slightly lower TOC removal ratios than the Coagulation–Membrane rotation for the SA solution under membrane rotations, indicating that the coagulation could effectively aggregate the macro molecule organic matters to form large flocs to settle with a low membrane rotation speed, resulting in a high organic removal. While in the TA solution, the organic removal with 30 r/min membrane rotation was lower than the Coagulation–Membrane rotation, suggesting that the coagulation was less effective in micro molecule organics than in macro molecules. Dong et al. studied the coagulation–microfiltration process for NOM removal, and they also found a similar performance that the coagulation was more effective for macro organic substances removal and less effective for micro organic molecules removal [43]. With coagulation, the membrane rotation had a much higher TOC removal than the direct membrane rotation without coagulation in both SA and TA solutions (Figure 5), suggesting that Coagulation–Membrane rotation was effective for DOM removal.

**Figure 9:**
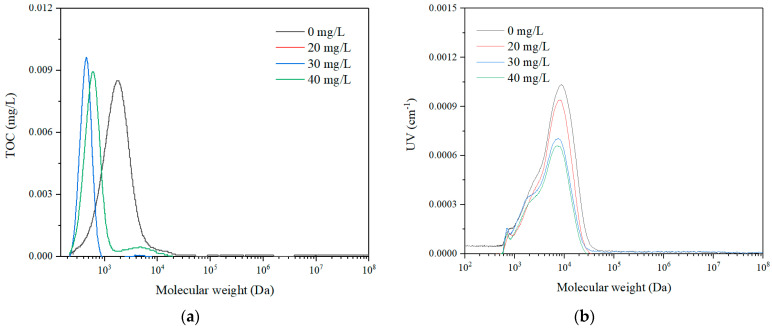
Molecular weight distribution of SA with a rotation speed of 30 r/min and different coagulant dosages, (**a**) TOC detection, 3 h, and (**b**) UV detection, 2 h.

The molecular weight distribution of SA solution at a rotation speed of 30 r/min with different dosages of coagulants was examined and the result is exhibited in Figure 9. With the increase of coagulant dosages, the molecular weight distribution of the permeate SA solution shifted from the macro molecules to the micro molecules in the TOC detection, which was probably due to the fact that the coagulants could aggregate the macro molecules to retain on the membrane surface while allowing the micro molecules to permeate the membrane. The molecular weight distribution in the UV detection showed that the intensity of the macro molecules decreased greatly, which was consistent with the TOC detection result that the macro organics were significantly removed with the Coagulation–Membrane rotation process. Comparing with the molecular weight distribution of the permeate in the membrane rotation system without coagulation (Figure 5 and Figure 6), the organics with coagulation and rotating hollow fiber treatment have smaller particle size with less intensity, indicating that the combined process is effective for mediate and large molecule organics removal to remain fine organics in the effluent.

#### 3.2.2. Effect of Rotation Speeds on Coagulation–Membrane Rotation Performance

**Figure 10:**
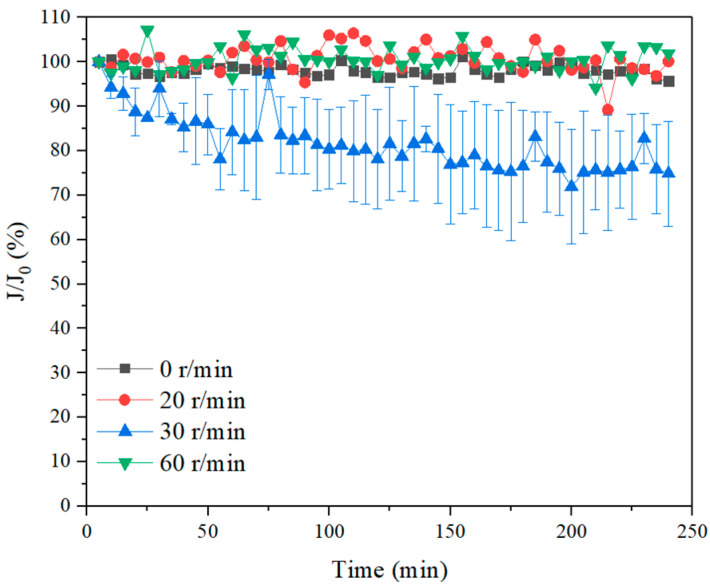
Permeate flux with 30 mg/L coagulant and different rotation speeds in SA solution.

The permeate flux with different rotation speeds and 30 mg/L PAC dosage were investigated, with the result presented in Figure 10. Without membrane rotation, the permeate flux maintained nearly constant, which was likely due to the fact that the coagulants could aggregate the organics to become large flocs to settle, resulting in low membrane fouling and stable permeate flux. When 20 r/min membrane rotation was applied, the permeate flux remained unchanged. When the membrane rotation speed increased to 30 r/min, the flux dropped significantly, which was probably attributed to the large rotation speed being able to generate high shear stress that could break the flocs resulting in the pore blockage of membrane filtration mode and the deterioration of the membrane permeate performance. The particle size of the flocs with different rotation speeds is discussed in detail in the next section. When the rotation speed further increased to 60 r/min, the flux remained constant, suggesting that strong membrane rotation could induce not only high shear forces, but also vortex, thus mitigating membrane fouling. With the elimination of the foulants with the combined process of coagulation and membrane rotation, the filtration lifetime of the membrane was prolonged. However, the risk of fiber breakage also heightened at the same time due to the continuous stress on the fibers during rotations. The damage usually occurred at the potting of the fibers, which was also a great concern in submerged membrane bioreactors with repetitive cyclic stresses with transverse vibrations [21,23]. Further investigation in the fiber breakage potential is therefore demanded before prototype implementation.

**Figure 11:**
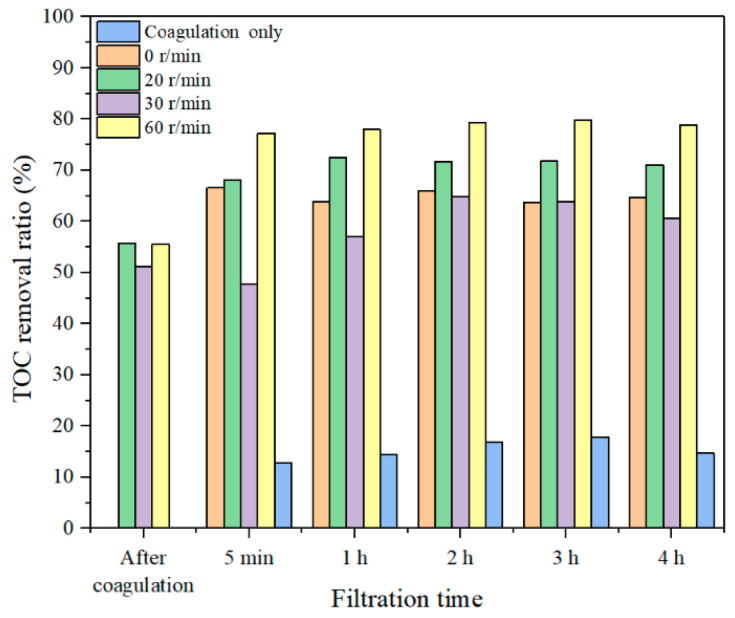
TOC removal ratios with 30 mg/L coagulant and different rotation speeds in SA solution.

The TOC removal ratio for the SA solution with 30 mg/L coagulant and different membrane rotation speeds were investigated and the result is shown in Figure 11. The direct coagulation could remove ~15% organic substance. While with fiber rotation, the organics removal ratios could be greatly enhanced. For example, with a 20 r/min rotation and 30 mg/L coagulant dosage, the TOC removal ratio was nearly 70%, while it increased to above 80% when the membrane rotation speed increased to 60 r/min, which was due to the generated high shear stress prevented the buildup of organics on the membrane surface and reduced the organics mass transfer. Such observations were also examined in river water and industrial water treatment with coagulation combined membrane vibration processes [44,45]. It is noticed that there was a low TOC removal ratio when the rotation speed was at 30 r/min, probably due to that the flocs were broken down at such rotation speed and some micro molecules passed the membrane pores resulting in a decreased TOC removal ratio. While the high organics removal with combined coagulation and 60 r/min membrane rotation was probably due to the generation of high shear stress and vortex that obstructed foulants approaching membranes. The TOC removal ratios exhibited quite stable after 5 min filtration, suggesting that the Coagulation–Membrane rotation was effective for long-time organic removals.

**Figure 12:**
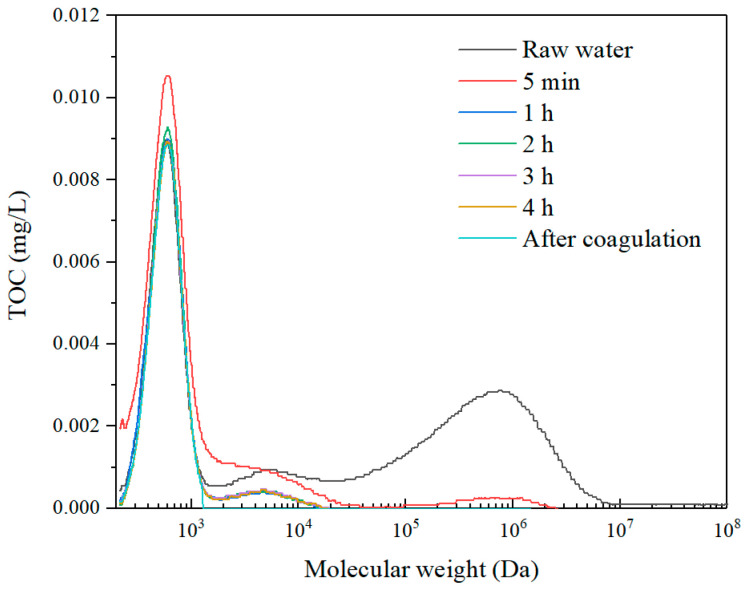
Molecular weight distribution (TOC detection) with a rotation speed of 30 r/min and 20 mg/L coagulant in SA solution.

The molecular weight distribution of the SA solution, direct coagulation and the permeate for different sampling intervals were investigated with the results shown in Figure 12. It can be observed that the peak of the macro molecule organics of SA with the molecular weight of 100,000–1,000,000 Da dropped fast after the coagulant was dosed. With the UF membrane, the peak of the macro molecule organics with a molecular weight of 100,000–1,000,000 Da and the mediate molecule organics with a molecular weight of 10,000–100,000 Da decreased first, then the peak of the macro organics completely disappeared, and the intensity of the medium molecules reduced significantly. Comparing with the molecular weight distribution of SA with only the membrane rotation (Figure 9), the organics with the combined process have smaller molecular weights. Thus, it can be revealed that the combination of coagulation and membrane rotation was very effective for the removal of not only macro organic substances but also medium organic matters.

#### 3.2.3. Particle Size Distribution

**Figure 13:**
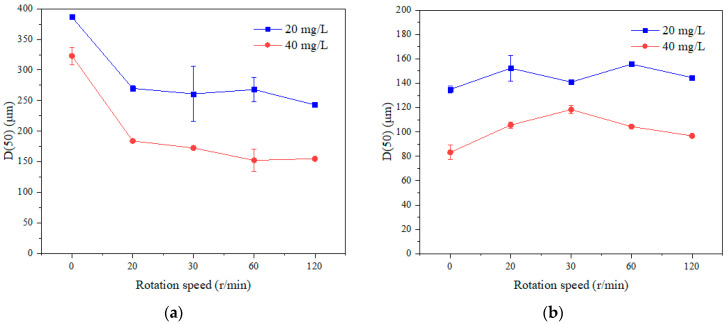
Average particle size of the flocs with different dosages of coagulants and rotation speeds, (**a**) SA, and (**b**) TA.

To further investigate the effect of coagulation - membrane rotation on the membrane filtration performance, the particle size distribution of both SA and TA with different concentrations of coagulants and rotation speeds were examined in triplicate with the results shown in Figure 13. Generally, the SA has a much larger particle size than the TA no matter how many coagulants were dosed or rotation speeds were applied. The particle size distribution of SA decreased from 386.5 ± 2.1 µm to 243 ± 1.4 µm and TA remained stable, ~140 µm when the rotation speed was increased from 0 to 120 r/min with a coagulant dosage of 20 mg/L. Similar results were also obtained when the 40 mg/L coagulant was added. For both solutions, a lower dosage of 20 mg/L coagulant induced a larger particle size than the 40 mg/L dosage. Thus, the different membrane filtration performance of SA and TA was probably due to the fact that the SA has a higher molecular weight (Figure 2) and larger particle size, and it was easy to aggregate to form large and loose flocs with the coagulation; with the membrane rotation, the fluid in the reactor was mobilized, and large and loose flocs were broken to smaller ones. While the TA has a lower molecular weight (Figure 2) and smaller particle size, and it formed smaller flocs with the coagulation, and the membrane rotation has a less destructive effect on the smaller size flocs. With too many coagulants, the coagulation effect became less effective and smaller flocs were formed. Li et al. pointed out that smaller particle size induced lower shear stress in a vibratory shear enhanced system [20,23]. Ma et al. investigated the membrane rotation and coagulation with Al-based coagulant for HA removal, and found that the floc-based cake layer could be broken by strong shearing forces, making HA molecules directly reach the membrane surface to further aggravate membrane fouling [46]. While in our system, the smaller flocs induced less shear stress with the membrane rotation, and they may form a smoother cake layer resulting in a lower organic removal as compared with the larger flocs.

#### 3.2.4. Membrane Surface Morphology Examination

**Figure 14:**
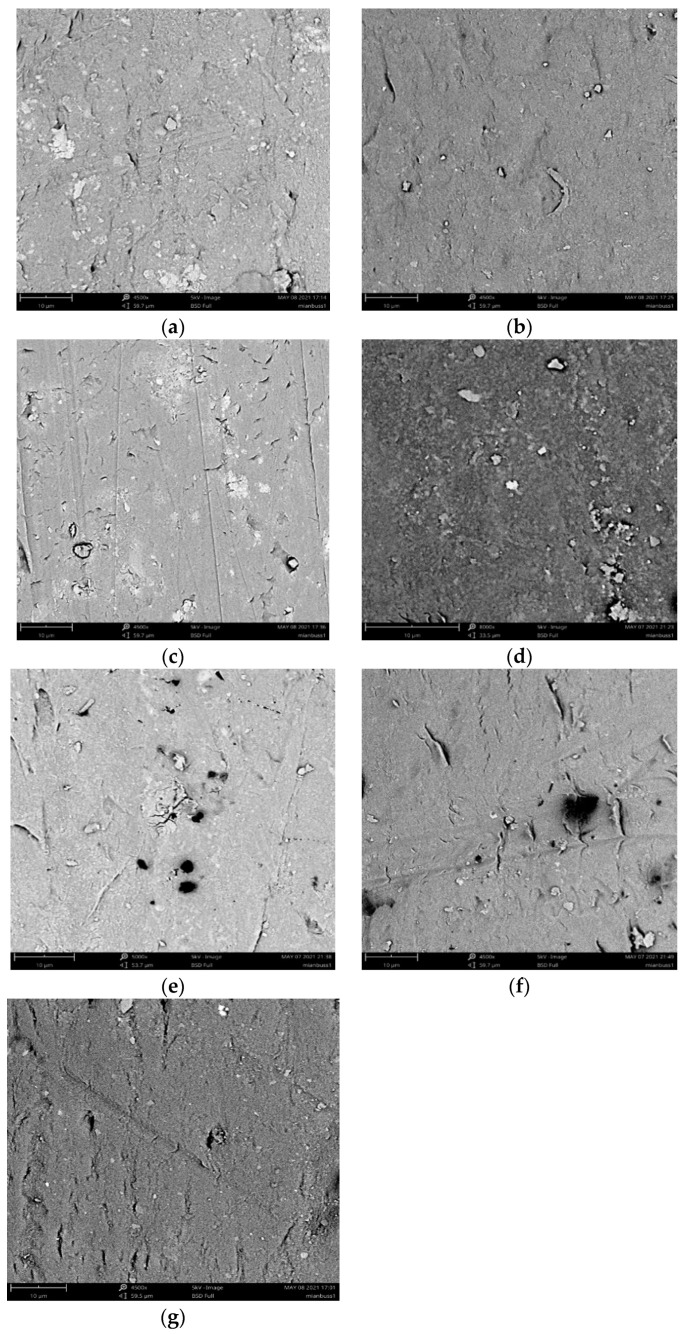
SEM images of hollow fibre membrane with SA or TA filtration with different rotation speeds and coagulation, (**a**) SA, 0 r/min, (**b**) SA, 60 r/min, (**c**) SA, 60 r/min with coagulation, (**d**) TA, 0 r/min, (**e**) TA, 60 r/min, (**f**) TA, 60 r/min with coagulation, and (**g**) blank membrane.

The surface morphologies of the hollow fiber membrane with SA or TA filtration with different dosages of coagulants and rotation speeds were examined and the results are exhibited in Figure 14. With membrane filtration, the organics were retained by the fibers, which can be directly detected from the SEM images. In the direct filtration, there were more SA organic molecules than TA organics on the membrane surface, indicating the membrane could retain more macro organics. With 60 r/min membrane rotation, the deposition of both SA and TA molecules on the membrane surface was reduced, suggesting that the membrane rotation could prevent the buildup of organics on the membrane surface, thus reducing membrane fouling. While with additional dosage of 20 mg/L coagulant, the deposition of organics on the membrane surface became even less, but the size was increased. Thus, with the effect of the coagulation, large flocs could form, while the fiber rotation can sweep the foulants efficiently. These observations further suggested that the coagulation and membrane rotation could greatly reduce membrane fouling and enhance organic removal.

## 4. Conclusions

In this study, the combination of coagulation and membrane rotation was applied for the fouling control and organic removal of dissolved organic matter (DOM), including macro molecule organics, i.e., sodium alginate (SA), and micro molecule substances, i.e., tannic acid (TA). The results indicated that the membrane rotation was very effective for fouling mitigation of both macro and micro molecules to prolong membrane filtration lifetime, while it had a high organic removal in SA solution than in TA solution through rejecting the large molecule organics. With the membrane rotation speed of 60 r/min, the permeate flux and the organic removal increased 90% and 35% in SA solution, respectively, as compared with the membrane performance without fiber rotation. With additional coagulation, the permeate flux improved due to the aggregation of organics into flocs and the subsequent retention by the rotating membranes, e.g., additional 70% flux enhancement and 5% organic removal increment to 80% were obtained with 40 mg/L coagulant added at the fiber rotation speed of 60 r/min. The high membrane rotation speed could induce large shear stress and vortex for fouling control, however, too much rotation would heighten the risk of fiber damage, break the flocs and result in the reduction of permeate flux and deterioration of permeate quality. With the combined effect of coagulation and fiber rotation, the cake layer on the membrane surface was significantly reduced. Therefore, the Coagulation–Membrane rotation is a very promising technology for drinking water treatment, and it can be further explored in the future.

## Figures and Tables

**Table 1:** Product information of SA, TA, and PAC used in the present study.

No.	Name	Molecular Formula	Manufacturer
1	Sodium alginate	(C_6_H_7_NaO_6_)*_n_*	Sinopharm Chemical Reagent Co., Ltd. (Shanghai, China)
2	Tannic acid	C_76_H_52_O_46_	Sinopharm Chemical Reagent Co., Ltd. (Shanghai, China)
3	Polyaluminum chloride	AlClHO	Xiya Reagent (Chengdu, China)
